# Hydromechanical Structure of the Cochlea Supports the Backward Traveling Wave in the Cochlea *In Vivo*

**DOI:** 10.1155/2018/7502648

**Published:** 2018-07-17

**Authors:** Fangyi Chen, Dingjun Zha, Xiaojie Yang, Allyn Hubbard, Alfred Nuttall

**Affiliations:** ^1^Department of Biomedical Engineering, Southern University of Science and Technology, Shenzhen, Guangdong 518055, China; ^2^Oregon Hearing Research Center, Department of Otolaryngology and Head and Neck Surgery, Oregon Health and Science University, Portland, OR 97239, USA; ^3^Department of Otolaryngology-Head and Neck Surgery, Xijing Hospital, Fourth Military Medical University, Xi'an, Shaanxi 710032, China; ^4^Department of Electrical & Computer Engineering, Boston University, Boston, MA 02215, USA; ^5^Kresge Hearing Research Institute, The University of Michigan, Ann Arbor, MI 48109, USA

## Abstract

The discovery that an apparent forward-propagating otoacoustic emission (OAE) induced basilar membrane vibration has created a serious debate in the field of cochlear mechanics. The traditional theory predicts that OAE will propagate to the ear canal via a backward traveling wave on the basilar membrane, while the opponent theory proposed that the OAE will reach the ear canal via a compression wave. Although accepted by most people, the basic phenomenon of the backward traveling wave theory has not been experimentally demonstrated. In this study, for the first time, we showed the backward traveling wave by measuring the phase spectra of the basilar membrane vibration at multiple longitudinal locations of the basal turn of the cochlea. A local vibration source with a unique and precise location on the cochlear partition was created to avoid the ambiguity of the vibration source in most previous studies. We also measured the vibration pattern at different places of a mechanical cochlear model. A slow backward traveling wave pattern was demonstrated by the time-domain sequence of the measured data. In addition to the wave propagation study, a transmission line mathematical model was used to interpret why no tonotopicity was observed in the backward traveling wave.

## 1. Introduction

Ears not only hear sound but also generate sound, which is called the otoacoustic emission (OAE) and was discovered in 1978 [1]. There are currently two competing theories established to explain the propagation of sound from the place where it is produced inside the cochlea towards the exit of the cochlea. The backward traveling wave theory, which postulates that OAE-induced waves travel slowly along the basilar membrane (BM), is widely accepted as an explanation of the propagation of the OAE [1, 2]. However, this predominant backward slow-wave theory cannot explain some experimental phenomena [3–6] favoring the fast compression wave theory that would exist in the lymph fluids surrounding the BM. In the compression wave theory, the slow-speed propagation of the backward transversal wave motion of the BM is replaced by a fast-fluidic compression wave and experimental time/phase differences are accounted for by mechanisms independent of the wave. To date, it is still an open question about the OAE path. Resolving this question is important because the OAE has become a useful and noninvasive clinical tool for hearing screening. To utilize the OAE for more precise diagnoses, it is necessary to understand how it propagates backward to the ear canal. In relation to the backward propagation of the OAE, von Békésy discovered that the wave on the BM always traveled from the base to the apex, even the stimulus (the stapes vibration) was placed at the apex of the cochlea. This so-called paradoxical wave was a hurdle that prevented people from believing that the OAE can propagate along the BM backward at the time when OAE was just discovered. In a few years since then, the seeming contradiction to the paradoxical phenomena was explained by stating that the vibration source of OAE was on the BM rather than in the fluid by the stapes as in the paradoxical wave. Hence, the theory of backward traveling wave was widely accepted.

One critical issue that causes the uncertainty in determining the wave propagation mechanism is that the actual location of the OAE source is uncertain. Most of the previous studies used nonlinear, intrinsic vibration to the organ of Corti to generate organ vibration at the intermodulation frequency of two tones. This intrinsic vibration initializes the backward wave propagation, but the spatial location of the vibration along the BM is still debated. This caused the analysis of the experimental data to be difficult because the generation mechanism of the OAE is complicated [2], and the origin of the OAE could be multiple sites. Although the width and location of the generation site are so important, they cannot be ascertained. The uncertainty of the source location also makes it difficult to estimate the wave propagation speed, which is a critical data for distinguishing the propagation forms and testing which theory explains the sound propagation mechanisms. Such an uncertainty has often resulted in interpretations of the same experimental results [6–8] using both competing theories. Thus, it is critical to study the backward wave propagation from a vibration source at a unique and precise location in the cochlea.

In addition, although as a widely accepted theory, the backward traveling wave theory has not been validated directly using an experiment where the vibration on the BM is measured [3]. The evidence used to support this theory is mostly from mathematical modeling [7, 9–14] and indirect measurements [15, 16]. Attempts have been made to experimentally generate local vibration on the BM via optical [17] and direct mechanical [18] methods but with limited success. The optical method [17] does not provide enough mechanical vibration until the light is strong enough to cause damage to the organ of Corti. The mechanical stimulation in Richter et al. [18] did provide an accurate vibration source with enough energy, but the hemicochlea preparation in this study destroyed the integrity of the basic hydromechanical structure of the cochlea. Also, the poor sensitivity (~10 nm) of the vibration measurement method limited its capability to detect the small vibration on the BM.

Since the hypothesized backward traveling wave is a transverse vibration on the BM, these indirect measurements [15, 16] have also been criticized to not truly represent the BM vibration [19, 20]. Moreover, other structures (e.g., tectorial membrane and Reissner's membrane) in the cochlea have also been suggested to be capable of supporting wave propagation inside the cochlea [21, 22], but their contribution to the cochlear mechanical dynamics has not been thoroughly studied and determined. Therefore, a direct measurement of the BM vibration using an experiment is necessary to determine the wave propagation mechanism of OAE in cochlea.

In the present study, we designed a novel method using a mechanical/piezoelectric stimulator to drive the BM at a precisely known location; thus, the location of the vibration source initiating the backward wave was accurately known. The BM vibration was then directly measured at multiple locations in an *in vivo* cochlear preparation. We found that the phase of the measured BM motion showed a consistent lag with the increase of the distance from the vibration source. The speed of the wave propagation was shown in the order of tens of meters per second, much less than the speed of a compression wave in water. This study, for the first time, used directly an experiment to prove that there was a slow backward traveling wave on the BM of the cochlea *in vivo*. The computer simulation of the experimental results in a mechanical model, termed “artificial cochlea,” also confirmed that the hydromechanical structure of the cochlea supported the backward traveling wave.

## 2. Methods

### 2.1. Specimens

**Figure 1:**
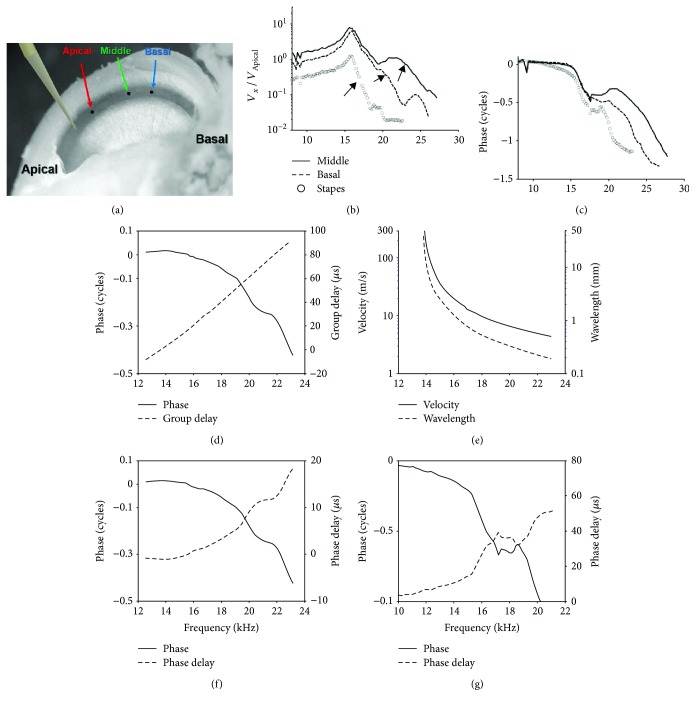
BM and stapes vibration spectra induced by a point stimulation of the BM. (a) The opened cochlea with stimulator and reflective beads in place. The stimulator probe is a blunt pipette driven by a piezoelectric stack. Reflective beads are placed on the BM, labeled as apical, middle, or basal, in the direction away from the stimulator. A bead was also placed on the stapes for recording its vibration. Vibration amplitude (b) and phase (c) of the middle (solid), basal (dash), and the stapes (circle) beads, relative to that of the apical bead. (d) Phase difference (solid) between the middle and basal beads and the calculated group delay (dash). (e) Wave velocity and the wavelength, calculated from the phase difference in *D*. The negative delay value at lower frequencies (<14 kHz) in both (d) and (e) is likely due to a local disturbance, such as the local reflection with the insertion of the stimulator. The wave speed is high at lower frequencies, and the distance between these two beads is small. Both effects make it vulnerable to the disturbance. For a wider range, apical to stapes, this small negative delay is absent (g). (f) Phase difference (solid) between the middle and basal beads and the calculated phase delay (dash). (g) Phase of the stapes vibration (solid) relative to the apical bead and the calculated phase delay (dash).

A total of 6 young guinea pigs weighted at 250 g–400 g were used in this study. After an animal was anesthetized by intramuscular injection of a mixture of ketamine (30 mg/kg) and xylazine (20 mg/kg), the temporal bone was opened using the dorsal-ventricle approach as described by Zheng et al. [23]. During the experiment, the animal was anesthetized with regular supplements of anesthetics. Tracheotomy was performed, and a ventilation tube was inserted into the trachea for natural breathing. The guinea pig's head was then mounted on a heated head holder. A surgical operation was performed to expose the left bulla, which was then opened for access to the cochlea. During the experiment, the core temperature of the animal was maintained at 37°C-38°C by a heating blanket and a rectal thermometer, which was controlled by a servo temperature controller (FHC, Bowdoinham, ME, USA) [23]. After the bulla was opened to expose the cochlea, an oblong window of about 2 mm long and 0.5 mm wide was opened on the bony wall at the cochlear basal turn to expose the BM. As shown in Figure 1(a), the basal turn of the cochlea was cut to open and the BM was exposed over almost the whole basal turn. Reflective beads assigned names of basal, middle, and apical were placed on the BM along the longitudinal direction. The tip of stimulator touched the BM at the apical end of the basal turn and delivered the vibration to initiate the backward wave. The wave propagation along the BM was recorded by focusing the laser of the vibrometer onto the reflective beads.

In the preparation, the joint between the incus and stapes was dislocated so that the middle ear chain was disrupted, significantly reducing any possible middle-ear-conducted acoustic stimulation. The ossicular dislocation was done because the piezo stack used to stimulate the BM also radiates acoustic energy. This sound will propagate to the adjacent tympanic membrane, initiating a normal acoustic stimulus to the cochlea and thus producing a forward traveling wave in the cochlea if the ossicular chain is intact. That forward traveling wave would interfere with the backward wave initiated by the stimulation probe on the BM. The fluid level of the perilymph in the scala tympani was carefully lowered using cotton wicks so that only a very small amount of fluid (~30 *μ*m thick based on visual comparison with the known diameter of the probe tip) was left to moisten the exposed BM.

The study was approved by the Institutional Animal Care and Use Committee of Oregon Health and Science University.

### 2.2. Piezo Stimulator Design and Mounting

**Figure 2:**
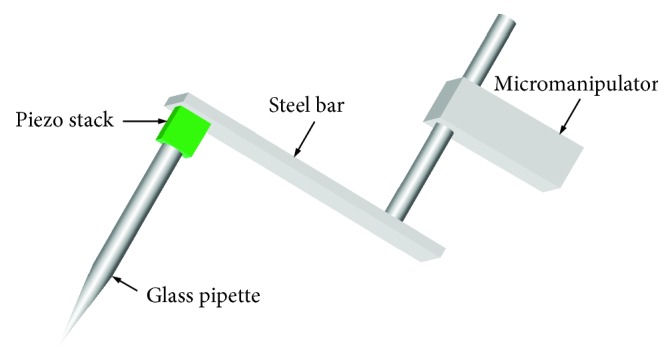
Construction of the piezo stimulator. Glass pipette with melted tip was glued on a piezo stack and then on a steel bar, which was fixed on a micromanipulator.

The accurate location of the vibration source was a problem in almost all of the previous studies designed to measure the backward wave propagation in the cochlea. In our study, the direct mechanical driving method was adopted with a different design. The tip of a pulled pipette when heated with an electrical cautery, melted into a sphere of about 50 *μ*m in diameter. This rounded tip was appropriate in size compared to the BM width for the delivery of a local vibration. Then a ~2 cm piece from the tip end of the pipette was cut off and attached to a piezo stack (AE0203D04F from Thorlabs, Newton, New Jersey) using cyanoacrylate cement. The driver was then cemented onto a steel bar, which was mounted on a micromanipulator.  Figure 2 shows a view of the assembled probe. The overall size of the piezo stimulator allowed it to fit under the objective lens of the laser Doppler vibrometer (LDV). During the experiment, the piezo stimulator was advanced in about a 60-degree (relative to the horizon) angle by the micromanipulator into the opened scala tympani and to get contact with the BM by the tip. This process is visually guided under a surgical microscope. Careful attention was taken to avoid significant deformation or perforation on the BM. After placing the stimulator, the surgical microscope was moved away and the LDV with the objective lens was then moved in for vibration measurement. The piezo stack and the steel bar were held in such a way as to reduce interference caused by the laser beam of the velocimeter. The contact of the probe tip to the BM was also verified by measuring the vibration of the bead that is closest to the probe on the BM.

### 2.3. Calculation of the Delays

Two kinds of delays, group delay and phase delay, were calculated from the phase spectra in this study. The group delay is the derivative of the phase difference. To avoid the influence of the noise, the phase data was firstly fitted with a 4th-order polynomial to smooth the curve before performing the differentiation.

(1) $$\begin{array}{c} Group\;delay=-\frac{d\varphi }{2\pi df}, \end{array}$$

where *d* is the differential operator, *φ* is the phase in radius, and *f* is the frequency.

Group delay has been used in most OAE studies [2, 5, 15, 16, 24] to quantify the delay for determining the direction of the wave propagation.

Besides group delay, another method to quantify the delay from the phase response is to calculate the phase delay by

(2) $$\begin{array}{c} Phase\;delay=-\frac{\varphi }{2\pi f}. \end{array}$$

This method has been adopted in previous studies by Ren and his colleagues [3–6, 17, 19].

### 2.4. The Mechanical Cochlear Model

In this study, a mechanical model of the cochlea was used to verify the backward wave propagation. This mechanical device consisted of a fluid channel, a membrane section, and an artificial BM to simulate the basic hydromechanical structure of the cochlea [25]. The artificial BM was constructed from a polymer membrane with 32 copper beams deposited on it. The lengths of the copper slots increased gradually from one (basal) end to the other (apical) and resulted in a stiffness gradient for simulating that in the cochlea. Vibration measurement on this mechanical model demonstrated the traveling wave-like features, that is, tonotopicity, the frequency-to-place map, unsymmetrical filtering (the shallow slope at the low-frequency side of the peak but steep slope at the high-frequency side), and slow traveling wave phase. Therefore, it is a valid model of the cochlear hydromechanical structure for verifying the backward wave propagation.

### 2.5. Transmission Line Model of the Cochlea

**Figure 3:**
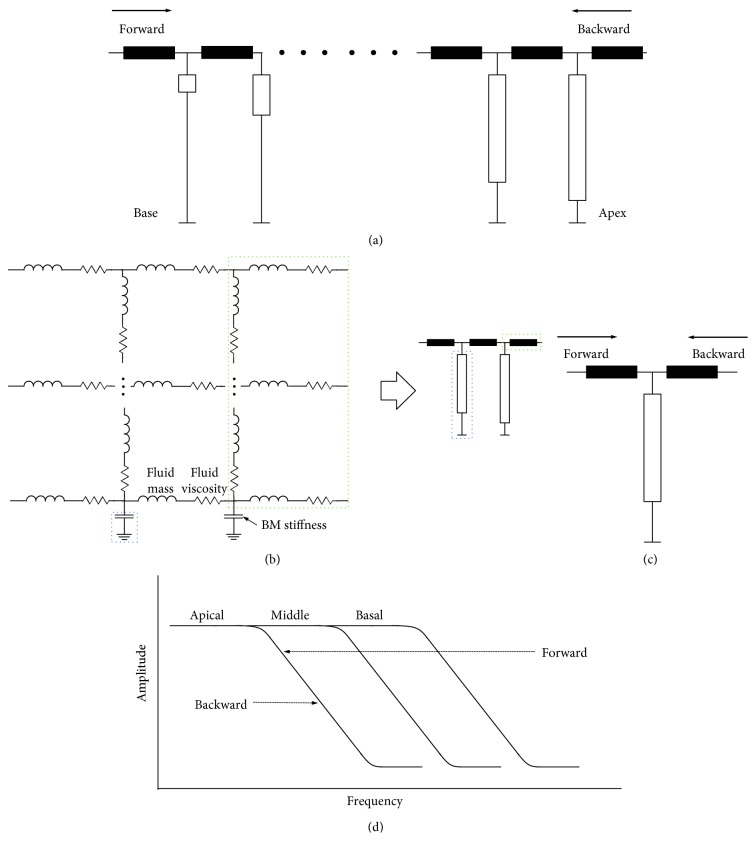
Model of the cochlear passive hydromechanical structure. (a) Schematic of the model structure. The thick solid lines represent the fluid channel and the open rectangles represent the cochlear partition, or simply BM. In this lumped model, the fluid channel and the cochlear partition (or simply BM) were divided into a series of sections. The rectangle is wider and shorter at the base and thinner and longer at the apex, representing the BM stiffness gradient: stiffer at the base and softer at the apex. (b) Circuit analogy of each section. The fluid mass is analogous to a series of inductors; the fluid viscosity is analogous to a series of resistors; the stiffness of the partition is analogous to a capacitor. (c) At each section, the forward and backward waves experience the same low-pass filtering. (d) Schematic shows the low-pass filters that the traveling wave experiences during the forward and backward propagation. Note that the forward direction is towards the low frequency. The roll-off frequency at the basal, middle, and apical locations decreases.

A mathematical model, lumped transmission line model, was created and simulated in a circuit simulator LTSPICE (Linear Technology, Milpitas, CA) based on acoustic-electrical analogy. The basic structure of the model is shown in Figure 3(a) and reviewed by Ni et al. [26]. This circuit model was firstly developed by Peterson and Bogert [27] and represented the basic hydromechanical property of the cochlea. Rather than a one-dimensional structure, our model includes a two-dimensional matrix of mass and dampers to represent the fluid channel. The cochlear partition was modeled as 400 sections of dampened mass-spring resonators and coupled with the fluid channel. No active component (outer hair cell) was included since the experimental preparation was passive. By placing a vibration source at the very basal end of the fluid channel, we simulated the stapes driving the cochlea; by placing the source inside a more apical section on the BM, we simulated the backward wave case where the glass probe stimulates the BM directly. The BM stiffness was taken from Puria and Steele [28], and the cochlear tonotopicity was taken from Greenwood [29].

## 3. Results

### 3.1. *In Vivo* Data Demonstrated a Slow Backward Wave on the BM

For this experiment, the cochlea of a young guinea pig was surgically exposed and the basal turn of the cochlea was widely opened. Reflective beads were placed on the BM at different longitudinal locations and on the head of stapes, as shown in Figure 1(a). In a typical experiment, vibration spectra were measured from three beads (named apical, middle, and basal, according to their relative longitudinal locations) and from the stapes. Vibration spectra measured at the middle and basal beads were scaled by the spectra measured at the apical bead, the closest one to the vibration source, in order to calculate the transfer functions.  Figure 1(b) shows the amplitude responses. In this plot, the amplitude of all the transfer functions demonstrated peaks at about 16 kHz and a gradually decreasing roll-off frequency in the direction away from the stimulus. This peak is likely due to the standing wave that was introduced by inserting the probe. In absolute units, the maximum vibration velocity magnitudes are 1.3 mm/s, which correspond to a displacement of 12 nm at the 16 kHz peak of the middle location response. As indicated by the arrows in Figure 1(b), in addition to the 16 kHz peak and the roll-off after, the amplitude responses showed higher-frequency roll-offs from about 23 kHz for the middle location to about 20 kHz for the basal location and about 17 kHz at the stapes. Corresponding to the gradually decreasing roll-off frequencies, the phase of the spectra demonstrates a significant phase lag from the middle to the basal bead and then the stapes, indicating a slow wave propagating backward in the cochlea. The phase lag from the apical location to the stapes is more than one cycle (Figure 1(c)).

Phase difference between two neighboring—middle and basal—beads was used to calculate values of the wave propagation parameters: delay, wave velocity, and wavelength. The phase data were smoothed and fitted with polynomial before calculating the delay and velocity, as stated in Methods. The group delay between the middle and basal beads gradually increased with an increasing vibration frequency. It amounted to 62 *μ*s at 20 kHz (Figure 1(d)). Correspondingly, the wave velocity and wavelength decreased. The wave velocity was about 6.4 m/s at 20 kHz for the distance of 400 *μ*m between the two beads (Figure 1(e)).

Another method used to estimate the delay from the phase response was to calculate the phase delay. In Figure 1(f), we show that the phase delay value also increases with increasing frequencies but was smaller than the group delay, about 10 *μ*s at 20 kHz. This resulted in a higher estimated speed of 38 m/s, as compared with using group delay. However, both speed values were much smaller than the estimated compression wave speed of approximately 1500 m/s. The calculated phase delay from the apical bead to the stapes is about 40 *μ*s at 20 kHz.

### 3.2. BM Vibrations from Conventional Acoustic Stimulation Validated the Preparation

**Figure 4:**
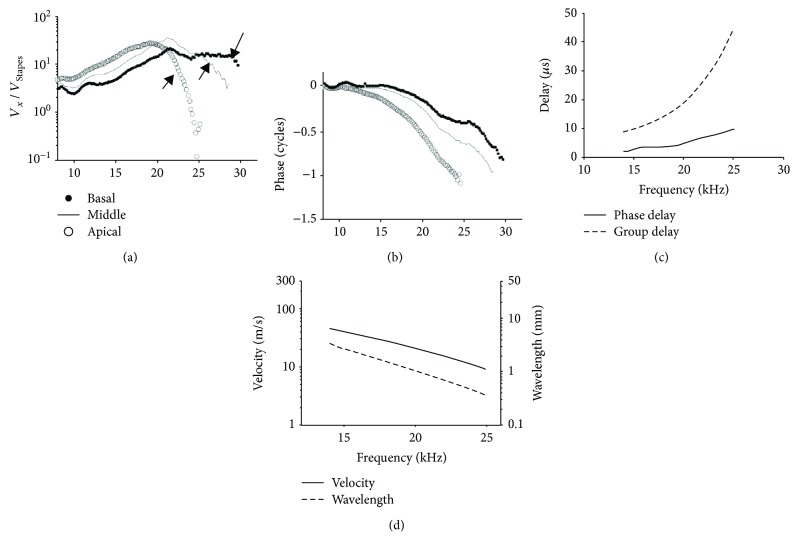
BM responses induced by normal acoustic stimulation at the ear drum. Vibration amplitude (a) and phase (b) of the beads on the BM. They are labeled as basal (dotted), middle (solid line), and apical (open cycle). Arrows in (a) indicates the roll-off of the amplitude responses. (c) Calculated phase and group delay between basal (dotted) and middle (solid line). (d) Calculated velocity (solid line) and wavelength (dashed line).

In this preparation, the sound-conduction system (i.e., the tympanic membrane and the ossicular chain) inside the middle ear was maintained to be intact and functional. The basal turn of the cochlea was widely opened, and the fluid at the scala tympani of the basal turn was maintained only at a level to moisten the BM. The cochlea thus became a single-channel hydrodynamic structure. To verify that this single-channel preparation preserved the forward traveling wave feature of the normal cochlea, the BM vibration was measured at the same longitudinal locations under normal acoustic conditions, that is, the sound was delivered to the ear drum. Rather than scaled by the vibration spectrum of the apical bead, here, the vibration spectra of three beads were scaled by the stapes vibration to obtain the forward cochlear-transfer functions. The amplitude plotted in Figure 4(a) shows a tonotopic map. The vibration spectrum rolled off at 28 kHz at the basal location; it rolled off at about 25 kHz at the middle location and at about 20 kHz at the apical location. Correspondingly, the phase plotted in Figure 4(b) shows gradually the increasing phase lag from the basal to the middle and then to the apical locations. These are expected traveling wave-like features under conventional acoustic stimulation. The phase and group delay increased with the increasing frequency (Figure 4(c)). The phase delay amounted to about 1/5 of the group delay, similar to what they were in the backward direction as shown in Figures 1(d) and 1(f). The velocity and wavelength decreased with the increasing frequency (Figure 4(d)). The velocity ranged from about 10 m/s to 40 m/s. Correspondingly, the wavelength ranged from 0.3 mm to 2 mm. The trend and the values of the delay, velocity, and the wavelength all indicated a slow forward traveling wave. This experimentally verifies the wave propagation function of the single-channel cochlea.

### 3.3. Forward and Backward Traveling Waves Were Observed in the Mechanical Model of the Cochlea

**Figure 5:**
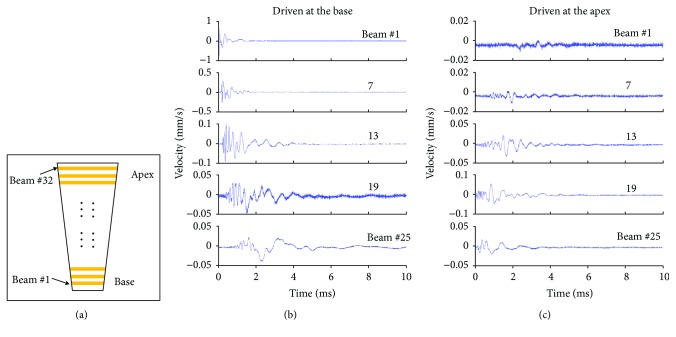
Vibration responses of a mechanical model of the cochlea. (a) Schematic view of the artificial BM. The length of the copper beam varies from 4 mm at the base (narrower) to 8 mm at the apex (wider). A total of 32 beams are placed on a membrane. The distance between beam #1 and #32 is about 20 mm. (b) BM vibration of the model while it was driven at the base, the narrowest, and thus the stiffest end. The driver was placed on the membrane right next to beam #1 at the base. Note that the scale of *y*-axis decreases from the top to the bottom, showing that the vibration amplitude was attenuated during the propagation. (c) BM vibration while it was driven at the apex. The driver was placed on the membrane at beam #29, close to the apex.

To better understand the role of the hydromechanical structure of the cochlea in the backward wave propagation, we studied a mechanical model of the cochlea. This artificial device was composed of a fluid channel to simulate the scala vestibuli and a membrane to simulate the BM.  Figure 5(a) shows the top schematic view of the device. Thirty-two copper beams are deposited on a membrane to form an artificial BM. The beam length varies gradually from beam #1 to beam #32, simulating the BM stiffness gradient from the base to the apex. The single fluid channel is underneath the BM (detailed construction of the device can be found in Chen et al. [25]). In a previous study, we showed that this hydromechanical structure could demonstrate cochlea-like features and its capability to support the forward cochlear traveling wave [25].

Figure 5(b) shows the time-domain response of the beam vibration, when the artificial cochlea was driven at the base, just before beam #1. About 1 ms delay was demonstrated between the onset responses of beam #1 and beam #25. The distance of these two beams was about 15 mm, so the speed of the wave propagation was about 15 m/s.  Figure 5(c) shows the results when the device was driven at the apex, on Beam #32. A clear backward delay was demonstrated in the plots. The amount of the delay was similar to its forward counterpart and so as the speed. This slow wave propagation implies that a backward traveling wave exists in this artificial device.

### 3.4. Simulation Results from a Mathematical Cochlear Model Where a Point Vibration Source Reproduced Features of the Experimental Data

**Figure 6:**
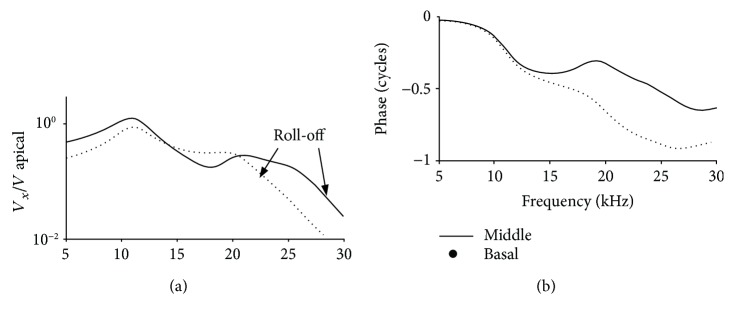
BM vibration responses of a mathematical model of the cochlea under backward driving. (a) Amplitude responses of BM vibration at two longitudinal locations. Solid line, labeled as middle, is at a more apical location; dotted line, labeled as basal, is at a more basal location. The arrows marked the roll-off points. (b) Phase responses.

In addition to the *in vivo* data and the experimental results from the mechanical model, we also studied the backward traveling wave in a mathematical model. This model has a traditional transmission line structure for simulating the hydromechanical structure of the cochlea. With this model, we simulated the backward responses. This was done by placing a vibration source at about 1/3 of the length of the cochlear model from the base and computing the BM vibration responses at more basal places towards to the stapes. BM vibration at three locations was computed to simulate the measured results at the apical, middle, and basal beads. As in the experimental results, the vibration spectra of the middle and basal beads were scaled by that of the apical bead, as shown in Figure 6. Features in the experimental results, as shown in Figures 1(b) and 1(c), were also demonstrated in the simulation results. The spectra can be viewed at two different frequency ranges. At lower frequencies (about <15 kHz), the amplitude response in Figure 6(a) shows a peak at about 12 kHz for both middle and basal locations. Correspondingly, the phase plotted in Figure 6(b) shows a steep roll off at the peak frequency. This could be due to reflection between the point source and the stapes. At frequencies lower than the peak, the phase responses of both locations almost overlap. This can be interpreted as a fast traveling wave at lower frequencies. The amplitude response rolled off at about 25 kHz for the middle location and at about 20 kHz for the basal location. Correspondingly, the phase response showed a clear delay from the middle to the basal location. This indicates a slow backward wave.

## 4. Discussion

### 4.1. Direction of the Wave Propagation

It has been hypothesized [1] that the OAE, generated as vibration at the organ of Corti, will propagate backward toward the stapes via a traveling wave on the BM. Although this theory is widely accepted and used to interpret the OAEs [2], the backward traveling wave has never been demonstrated with direct evidence of the BM vibration. Analysis of the OAE phase [9, 15] and cochlear microphonic signal that were measured at the round window [16] was used as indirect evidence of backward propagation. In this present study, the organ of Corti was set into vibration at a specific point location. This produced the first directly observed *in vivo* backward wave propagating on the BM. The phase plotted in Figure 1(c) has a gradual increase of phase lag at the vibration spectra measured at the apical (used as the reference; not shown) to the middle, the basal bead, and then the stapes. The velocity and delay calculated from the phase plot were within the same order of magnitude of their forward counterpart (Figure 4). These results are also evidence of a slow transverse wave propagating backward on the BM. In addition, we found a slow reverse wave propagation in a mechanical model of the cochlea (Figure 5). Since the cochlea prepared through the surgical procedure became a passive one (surgical damage removes the amplification by cochlear outer hair cells), this wave propagation appears to be a fundamental property of the hydromechanical structure of the cochlea. The artificial cochlea, which mimics the basic hydromechanical structure of the cochlea, confirms and supports the essential nature of the slow transverse waves, that is, they exhibit bidirectional propagation.

### 4.2. Interpretation of No Tonotopicity in Backward Wave Propagation

Although the experimental results in Figure 1 demonstrated the correct phase in reverse propagation, the amplitude response did not show the expected cochlear tonotopicity: the peak-frequency-to-place map [29]. In contrast, there was a slight inversion or reversal of the map, that is, the roll-off frequency is higher at the more apical location and lower at the more basal location. These seemly controversial reverse wave features are simulated by the transmission line model of the cochlea, as shown schematically in Figure 3.

The hydromechanical theory of the cochlea asserts that tonotopicity is achieved with the combination of fluid coupling in the scalae and a local resonance at the BM, the so-called critical-layer resonance [30]. For a passive cochlea, as used in this study, the hydromechanical structure is the dominant contributor [31]. The passive responses lack sharp tuning and diminish at high frequencies. Therefore, the local resonance is simplified in the model to a capacitor because the resonator is stiffness dominant at the lower frequencies. Acoustic energy in the fluid couples to the BM, whose stiffness decreases from the base to the apex.

Although the stiffness gradient is the most important feature for the tonotopicity [32], a small amount of frequency selectivity can be achieved without it. This process is most easily explained with an electric circuit analogy. The cochlear hydromechanical structure is analogous to a lossy transmission line, as originally proposed by Zwislocki [31]. In a lumped model, the cochlea is divided into a series of connected repeating sections (Figure 3(a)). At each section (Figure 3(b)), the fluid coupling effect was modeled as a series of inductors and resistors, representing the fluid mass and viscosity, respectively [33]; the mechanical impedance of the cochlear partition was modeled as a capacitor, representing the stiffness of the BM. Although the BM is usually modeled as a second-order resonator to account for the sharp tuning of the cochlea, generality is maintained with the simplification to a spring (capacitor in a circuit analogy) to model the passive responses. This simplification is fundamental to Zwislocki's transmission line theory [31] to interpret the passive vibration data observed by von Békésy [34]. With this simplification, the propagation of the BM vibration was described using a wave equation [31]. Each section of the structure applies a low-pass filtering effect onto the vibration of the BM, attenuating the high-frequency components and producing propagation delay. Vibration, originated from either a basal or apical location, will experience the same low-pass filtering sections (Figure 3(c)), but from different directions. Therefore, the high-frequency components of the BM vibration are continuously attenuated, and the phase delay is continuously accumulated during the propagation, no matter which end the wave originates.

Figure 3(d) explains why no cochlear tonotopicity is achieved in reverse propagation. In the forward direction, the stiffness of the cochlear partition decreases in the direction of the propagation. Therefore, the roll-off frequency of the low-pass filtering, implemented by the fluid mass and the stiffness of the partition, becomes lower and lower. Therefore, the high-frequency components of the traveling wave are cut off gradually during the propagation, resulting in the passive cochlear tonotopicity. In the reverse direction, the traveling wave encounters the low-pass section with the lowest roll-off first. Most of the high-frequency components are attenuated at this early section. During the propagation, although the traveling wave will still experience low-pass sections, its already low-passed components will not be further cut off because the roll-off frequencies of the later sections are all higher. The result is that no clear tonotopicity is produced, but the high-frequency components are still slightly attenuated along the direction of propagation.

Despite the significant difference in the amplitude responses, the transmission line-like structure of the cochlea produces a propagation delay in the direction of away from the vibration source, no matter where the vibration source is located. This is shown in both the experimental data in Figure 1(c) and the simulation results in Figure 6(b).

### 4.3. Validation of the Preparation

Since we opened the cochlea widely and drained the fluid in the scala tympani at the base, we need to ensure that this preparation can still support the wave propagation as a normal cochlea. The single-channel preparation for the measurement of basilar membrane vibration has been used previously [35, 36] and has theoretically been proven to be able to produce forward traveling wave features [37]. LePage [36] used a capacitive probe to measure the BM motion, which required the fluid in the scala tympani to be removed. It was shown that the BM vibration showed similar responses as its dual-channel counterpart. The acoustic driving results in Figure 4 also showed traveling wave features. Therefore, this preparation still maintains the basic hydromechanical structure of the cochlea. The *in vivo* physiological environment in the cochlea is maintained stably so that the mechanical properties provided by the cellular structure of the organ of Corti do not deteriorate during the experiments. Although this preparation is designed to verify the backward traveling wave, it does not preclude the existence of the compression wave, as the intact scala vestibule can still support the compression wave propagation. The open channel at the scala tympani could influence the speed of the compression wave. However, this influence is minimal considering that the speed of the compression wave is two orders of magnitude higher than that of the traveling wave. The BM vibration measurement method used in this study is also essentially the same as that used by He et al. and Ren et al. [3–5]. Therefore, if the compression wave does exist in this preparation, we should be able to observe its effect, as a transverse wave in the forward direction in our experiments.

To ensure that the vibration source in this study is on the BM, we drained the perilymph at the opened portion of the scala tympani and then maintained the fluid at a very low level (~30 *μ*m) with a cotton wick. According to the current theory, it is the vibration generated in the organ of Corti on the BM that initiates the backward traveling wave. The OAE is also generated in the organ of Corti. The stimulus in the fluid may result in a similar effect as the stimulation by the stapes, producing a forward wave. This phenomenon has been demonstrated by von Békésy [38] and termed as the *paradoxical wave*, where even if the stapes is placed at the apex of the cochlea, a forward traveling wave was still observed, moving towards the source.

### 4.4. Passive or Active Preparation

In this study, the extensive surgery on the cochlea resulted in a loss of sensitivity, so that the preparation was essentially passive. Although loss of cochlear amplification would influence the generation of the OAE, the passive preparation is an advantage in studying the wave propagation. The wave propagation inside the cochlea is mostly determined by the hydromechanical structure, and our preparation preserves the basic hydromechanical components of the cochlea.

The advantage in the use of the passive preparation is that it avoids any phase delay introduced by the active tuning of the cochlea. In a sensitive preparation, active tuning of the cochlea contributes strongly to the measured group delay [20]. Tuning-induced group delay is primarily responsible for the enhancement of the propagation delay in the forward-propagation condition, because the tuning frequency of the cochlea decreases in the forward direction and this frequency gradient produces a phase lag in the same direction. In a sensitive cochlea, artificially driven to produce a backward wave, tuning-induced forward delay may mask the possible backward traveling wave delay. The propagation delay increases while traveling away from the source. However, the tuning delay is determined by the cochlear frequency map and it always increases from the base to the apex, no matter where the source is. Therefore, in the measured phase responses in a backward-traveling-wave study, there are two components that can contribute oppositely to the total delay. This tuning-induced delay could be one way to explain the results of He et al., Ren et al., and Ren [3–6], in which the measured phase delay increased from the base to the apex and thus indicated the absence of a detectable backward traveling wave. In those experiments, however, a very sensitive preparation was usually required by the vibration-source-generation method (acoustically produced distortion product or electrically stimulated emission). The tuning delay may be significant, especially when the vibration at two close locations is compared to determine the direction of the wave propagation [3]. In their study, the measurement range was narrow, and thus, the propagation delay was relatively small, compared to the tuning delay.

## 5. Conclusions

In this study, we investigated the backward wave propagation inside the cochlea. By creating a vibration source at a precise location, we avoided the uncertainty of the place where the vibration was originated in most previous studies. With measurements at multiple longitudinal locations along the cochlea, we, for the first time, demonstrated a slow backward traveling wave towards the stapes. This result was also confirmed in a mechanical model of the cochlea and interpreted with a traditional transmission line mathematical model.

## Data Availability

The data used to support the findings of this study are available from the corresponding author upon request.
